# World Cup Challenges Are Not New: Increasing Complexity and the Evolution of Preparation Across 48 Years of FIFA World Cups

**DOI:** 10.1007/s40279-026-02466-9

**Published:** 2026-06-02

**Authors:** Alan McCall, Guilherme Passos-Ramos, Gary Lewin, Zoran Bahtijarević, Oscar Luis Celada, Nicklas Dietrich, Les Gelis, Ricard Pruna, Gregory Dupont

**Affiliations:** 1 School of Sport and Exercise Sciences, Edinburgh Napier University, Edinburgh, UK; 2 Arsenal Performance and Research Team, Arsenal Football Club, London, UK; 3 School of Sport, Exercise and Rehabilitation, University of Technology Sydney, Sydney, NSW Australia; 4 ⁠Brazilian Football Confederation, Rio de Janeiro, Brazil; 5 Endocrinology and Metabolism Laboratory, Department of Physiology and Biophysics, Federal University of Minas Gerais, Minas Gerais, Brazil; 6 Arsenal Women Football Club, London, UK; 7 Union of European Football Associations (UEFA), Nyon, Switzerland; 8 Medical Services, Spain Men’s National Football Team, Royal Spanish Football Federation (RFEF), Las Rozas de Madrid, Spain; 9 Medical Services, Atlético de Madrid, Madrid, Spain; 10 German National Team, Deutscher Fußball-Bund (DFB), Frankfurt, Germany; 11 Rome, Italy; 12 Medical Department, FC Barcelona, Barcelona, Spain; 13 Lille, France

## Introduction

“My biggest environmental challenge … was actually at a U20 World Cup in Nigeria in 1999. Our team was based near the Sahara desert, close to 40+ degrees Celsius … there were strong winds and sand coming in from the Sahara, razor sharp like sandpaper”.

While the 2026 FIFA Men’s World Cup is often framed as uniquely challenging, historical and experiential perspectives suggest that environmental and logistical stressors have always been integral to tournament football. One senior international medical doctor, with experience across five FIFA World Cups including semi-final and final appearances, recalled this youth tournament context where extreme heat and wind exposure contributed to nasal irritation and bleeding among players. While this example comes from a youth competition, often delivered with fewer resources and less external visibility than senior tournaments, it reflects a broader reality: teams across football have long been required to adapt to demanding and, at times, unpredictable environments.

The 2026 FIFA Men’s World Cup presents a combination of environmental and logistical challenges including heat, humidity, travel and altitude that are rarely encountered together within a single tournament [1]. What may distinguish 2026 is therefore less the presence of these challenges, and more their combination, variability and interaction across a geographically dispersed competition. These challenges are unlikely to be insurmountable; if anything, contemporary teams may be better positioned than ever to manage and potentially exploit such conditions. However, the application of evidence-based strategies in tournament settings remains variable, requiring flexible, context-specific and often individualised approaches in practice [2].

The perspectives presented in this article are informed by a series of informal interviews and discussions conducted within the author group and with additional contributors, including players and one of the world’s most experienced international football commentators. Collectively, the authors represent experience across FIFA World Cups from 1978 to 2022 and going into 2026, with individual experience that ranges from three to 12 tournaments. This includes playing and staff contributors with World Cup winning experience, as well as repeated semi-final and final appearances. In addition, several authors bring combined expertise across research and applied practice within both national and club team environments, integrating scientific and operational perspectives in elite international and club football. This breadth and depth of experience provides a unique applied perspective on how environmental and logistical challenges have been encountered, interpreted and managed across different World Cup contexts and insights into the 2026 tournament.

Importantly, these experiences illustrate how teams have long attempted to manage challenging environments, even in the absence of formalised scientific frameworks. In many cases, practice preceded science, with solutions emerging from necessity before being formally studied or validated. Even when evidence becomes available, its translation into real-world practice remains slow and inconsistent; it has been estimated that only around 14% of the best available evidence is adopted into routine prevention and treatment, typically taking close to 17 years to do so [3]. Reflecting on the 1999 tournament in Nigeria, the same medical doctor described how, despite limited scientific knowledge and resources at the time, he instinctively implemented strategies to manage the heat, noting that “some people called me crazy … I was telling everyone to wear suncream … we only drank water and salts”. This highlights how applied experience-driven solutions have historically underpinned preparation in demanding environments, often preceding and at times outpacing the formal development of evidence-informed approaches.

## World Cup Challenges Are Recurrent But Increasingly Complex and Evolving

The experienced international commentator reflected that each tournament has presented a distinct configuration of contextual challenges, extending beyond environmental conditions to include political, logistical and organisational factors. In earlier tournaments, these challenges were shaped by broader geopolitical contexts, recalling that in 1978 “we were advised not to go … it was a time of dictatorship”, while in 1982 the Falklands War influenced coverage and access.

Climatic stress has also been a recurring feature. The 1994 World Cup in the USA was characterised by high heat and humidity, which “definitely affected the football”, particularly with midday kick-offs. As the experienced international commentator described, “the game is fast and furious, the dehydration makes it not fast and not furious”. Notably, this observation was made in advance of much of the contemporary empirical work quantifying the relationship between environmental heat and match running performance. This experiential insight is consistent with more recent evidence demonstrating that higher environmental temperatures are associated with reductions in high-speed running and sprint activity during professional football matches [4].

A World Cup-winning player described the 1994 tournament as “his most challenging”, noting that “the heat and humidity just intensified within the match, not because it got hotter, but because with the context and pressures in the game you perceive it affecting you more and more … it’s the context of the game that’s changing”. This highlights how environmental stress is not experienced in isolation but may be shaped also by the evolving psychological and situational demands of tournament competition.

Logistical demands have also consistently shaped tournament experience. A senior medical practitioner noted that during the 2018 World Cup in Russia, “travel was more of the challenge … we were one of the teams that travelled the most kilometres”, while a head physiotherapist recalled that during the 2002 World Cup, “FIFA travel was organised for us, but it turned out not enough for the cargo we actually needed”. These examples highlight that even when infrastructure is provided, performance still depends on anticipating and managing operational detail specific to your team.

Less visible stressors are equally important. One medical practitioner emphasised the need to monitor “the epidemiological situation in the country where you are going”, recalling that “13 players and 6 staff ended up with norovirus over […] 12 h”. “[D]oing everything to prevent it in the first place is the key”. He further noted that this type of monitoring is undertaken at every tournament. Such risks are consistently present but vary depending on the specific context. Effective preparation requires not only awareness of these stressors, but an understanding of what is most likely to be encountered in each tournament environment.

Some dimensions of the performance environment have evolved. Advances in technology have improved monitoring and individualisation, while the rise of personal devices and social media has introduced additional layers of complexity. These social media platforms are now embedded in athletes’ daily lives, influencing how they engage with their performance environment. While they offer opportunities for self-representation and connection, they also expose athletes to ongoing scrutiny, feedback and, at times, abuse, with implications for well-being and behaviour [5, 6]. One performance director warned about a scenario at the 2026 tournament where there could be an electrical storm delaying a match for up to 2 h; “imagine if the players come back to the dressing room and go straight onto their phones scrolling social media to see what people are saying about them while waiting to go back on the field, we need to think about how to manage this, even if it likely won’t happen”.

Crucially, stressors rarely occur in isolation. Their defining characteristic is how they combine. As one performance director noted, earlier tournaments typically involved “one or two main factors”, whereas now in 2026 “it’s more a combination of challenges and how many at the same time”. From a physiological perspective, these interactions are non-linear and bi-directional: travel-related fatigue may impair recovery and exacerbate thermal strain, while heat exposure may disrupt sleep and contribute to cumulative fatigue.

Importantly, however, not all stressors are perceived negatively. As one world cup winning player noted, “for me the issue isn’t mental … you are excited, it’s not a problem, you’re playing at the World Cup”, highlighting that physiological and environmental demands may coexist with positive psychological responses. Indeed, one of the papers in this World Cup collection discusses mental health and creating nourishing environments for players and staff [7].

## Preparation and Planning Determine Performance

Across World Cups, the extent to which environmental and logistical challenges influence performance appears to be shaped less by the conditions themselves and more by the quality of preparation. Effective planning, prioritisation and execution are central in determining how teams respond to these demands [8].

As one senior international medical doctor emphasised, “everything is manageable” when supported by “good planning”. “[G]ood preparation is key … the day your team qualifies, you celebrate, but immediately you are then checking things”.

This process requires prioritisation. A performance director highlighted that “you have to pick the challenges you are going to address … sometimes you can’t hit everything”. While multiple stressors may be possible, focus should be placed on those most likely to be encountered, such as “heat and humidity and the travel”, while still maintaining contingency plans for less probable scenarios. Teams “plan to the final, with different pathways” and “put processes in place for various scenarios”, recognising that progression through the tournament may expose teams to different environments, travel demands and logistical constraints.

The importance of integration between departments has evolved. A head of performance described how in earlier tournaments “we didn’t have a health and performance team collaborating with logistics … they didn’t think much about how travel would impact fatigue and sleep”, resulting in players returning from matches at “5 or 6am” or even “7am”, that could have been avoided. In contrast, more recent approaches involve close collaboration, with “medical and performance working with logistics”, reflecting improved organisational alignment and planning.

Integration also extends beyond the national team environment itself. A senior club medical doctor highlighted the importance of communication, trust and continuity between clubs and national teams, particularly during major tournaments where players may arrive with accumulated physical and psychological load from long club seasons. A South American national team performance director further noted that “some players arrive mid-season, others after 60 games”, highlighting how players may enter tournaments in very different states of readiness depending on their domestic context. The senior club medical doctor noted that “effective preparation is not only about what happens during the tournament”, but also depends on “the preparation before arrival and the management of recovery and reintegration to their clubs afterwards”. “[T]he World Cup does not start when the player arrives in camp, and it does not finish with the final match”. … “[W]hen players return to their clubs after tournaments, understanding what they have experienced physically and emotionally can also be very important”. This reflects growing recognition of the importance of health and performance information exchange across football environments to support player readiness and continuity across the tournament cycle [8].

Preparation requires translating knowledge into practice. One medical doctor described delivering detailed guidance ahead of matches in extreme heat, including hydration and electrolyte strategies, noting that “we needed to be prepared … you can’t imagine how hot and humid it was”. At the same time, scientific recommendations must be adapted to real-world constraints, as “the science may say to stay” after matches, but “no one sleeps anyway … so we just get going asap”.

Preparation strategies may differ between individuals. Another World Cup-winning player described his preference not to simulate pressure in training, stating that “training should feel good … you want to go into the game calm and relaxed”, and that “you just can’t replicate the pressure in a match”.

Adaptation can provide competitive advantage. One player noted that familiarity with heat allowed his team to cope better than opponents, observing that “we were used to it … but they were suffering … they didn’t adapt well, you could see their faces turning red, they couldn’t run”. This reinforces the earlier point that environmental challenges are not inherently insurmountable. Rather, they may represent an opportunity for advantage when teams are better prepared, better adapted and more capable of responding to the specific demands of the environment.

## Fundamentals and Decision Making Anchor Performance

Resource constraints also influence preparation. As one performance director noted, “everything is manageable, but it also depends on the financial resources you have available”, affecting travel, accommodation and recovery environments. However, even in lower-resourced settings, the consistent application of fundamental practices such as hydration, sleep, nutrition, hygiene and routine remains both achievable and critical to supporting player readiness.

A senior international medical doctor emphasised that “most important is just to ask the player: how do you feel? How did you sleep? How did you eat?” adding that “no blood measure or technology has ever come close to these basics”. This reflects the continued importance of simple direct indicators of readiness, even within highly resourced environments [9]. In this context, another senior medical doctor emphasised that “the mental aspect is fundamental … we need to keep the heads fresh”, particularly during long tournaments.

Maintaining routine is critical. One highly experienced medical doctor recalled a situation where a minor deviation from normal practice resulted in a failure to provide water during a match, reinforcing the principle to “never change your normal practice … national team is not a place to change anything … minimise the changes”. This principle extends beyond medical and performance practices to broader aspects of daily routine. As one practitioner joked, “if a player usually eats a cheeseburger in the pre-match meal, you give him a cheeseburger”. While tongue in cheek, the comment  illustrates a broader principle; how maintaining familiar habits even in seemingly trivial ways can support comfort, reduce disruption and contribute to overall readiness within the tournament environment, even when such practices may not always align with standardised or evidence-informed recommendations.

Decision making is shaped by experiential knowledge, with practitioners often relying on intuition and experience alongside data when operating in fast-paced environments [10]. A senior physiotherapist highlighted that repeated exposure to tournaments leads to “organisational maturity and efficiency”, enabling teams to “cut through the noise” and focus on what matters most. He further reflected that the accumulation of experience across multiple World Cups allows staff to become more “savvy” in their preparation, not only in responding to environmental and logistical demands, but also in anticipating them.

This evolution reflects a broader shift in the performance landscape, characterised by expanded resources, increased use of technology and greater access to information. However, despite these advances, the fundamental challenge remains unchanged: the need to interpret complex, and often competing, information under time pressure. In this context, experiential knowledge developed through repeated exposure to tournament environments remains central to effective decision making. Teams with repeated exposure to World Cups may therefore develop a form of organisational “DNA”, whereby experiential learning accumulated over successive tournaments contributes to greater efficiency in preparation and decision making.

## Conclusion

The 2026 FIFA Men’s World Cup will expose teams to a complex combination of environmental and logistical stressors. However, these challenges are not new and may be better understood as the interaction of established stressors within a more variable tournament context.

Advances in scientific knowledge, technology and accumulated experiential learning have strengthened preparation capabilities. However, uncertainty remains inherent, and the translation of knowledge into practice continues to vary between teams and contexts.

In practical terms, effective performance management is unlikely to be achieved through isolated or prescriptive solutions. Instead, it requires the integration of scientific evidence with experiential knowledge, alongside the ability to apply both within the realities of tournament football.

The articles within this special World Cup collection aim to support this process by providing current evidence alongside applied perspectives, with a focus on translating knowledge into practice. In doing so, they also highlight the importance of aligning future research with the realities of tournament environments to enhance relevance and usability for practitioners. Ultimately, success is likely to depend less on the novelty or complexity of strategies, and more on the ability to prepare effectively, prioritise appropriately and execute consistently under pressure.

## Declarations

### Availability of Data and Material

No datasets were generated or analysed during the current article.
